# Mechanism of Shi-Yang-Xiao lotion in alleviating perianal eczema based on network pharmacology and experimental validation

**DOI:** 10.3389/fmolb.2025.1621398

**Published:** 2025-06-19

**Authors:** Chuyue Huang, Bingwen Zhou, Meng Duan, Tuotuo Zheng, Yan Tang, Qingrui Liu, Yujing Dong, Desong Kong, Qiang Leng, Lu Wang, Zhimin Fan

**Affiliations:** ^1^ Nanjing Hospital of Chinese Medicine affiliated to Nanjing University of Chinese Medicine, Clinical Innovation Center For Anorectal Diseases, Nanjing, Jiangsu, China; ^2^ Xuzhou Hospital of Traditional Chinese Medicine, Anorectal Department, Xuzhou, Jiangsu, China; ^3^ Chongqing Banan Traditional Chinese Medicine Hospital, Anorectal Department, Chongqing, China; ^4^ Nantong Hospital Affiliated to Nanjing University of Chinese Medicine, Anorectal Department, Nantong, Jiangsu, China; ^5^ Nanjing Hospital of Chinese Medicine affiliated to Nanjing University of Chinese Medicine, Science and Technology Department, Nanjing, Jiangsu, China

**Keywords:** perianal eczema, shi-yang-xiao lotion, network pharmacology, traditional Chinese medicine, JAK2-STAT3 pathway

## Abstract

**Background:**

Shi-Yang-Xiao lotion (SYXL), due to its anti-inflammatory and antipruritic properties, is widely used in treating Perianal Eczema (PE) with remarkable clinical efficacy. However, the chemical constituents of SYXL and its underlying mechanisms of action in treating PE remain to be elucidated.

**Aim of the study:**

To elucidate the molecular mechanisms underlying the therapeutic efficacy of SYXL in treating PE by integrating network pharmacology techniques with experimental validation.

**Materials and methods:**

Ultra Performance Liquid Chromatography–Quadrupole Time-Of-Flight–Mass Spectrometry/Mass Spectrometry (UPLC-QTOF-MS/MS) was utilized to identify the effective chemical constituents of SYXL. Potential targets and pathways were elucidated utilizing network pharmacology analysis. Subsequently, ELISA and Western blotting were utilized in a rat model of PE induced by DNCB, and in HaCaT cells co-stimulated with tumor necrosis factor-α (TNF-α) and interferon-γ for further validation.

**Results:**

UPLC-QTOF-MS/MS identified a total of 93 compounds. Network pharmacology analysis revealed that the Janus kinase (JAK)/signal transducer and activator of transcription (STAT) signaling pathway was predicted to play major roles in the therapeutic effects of SYXL on PE. *In vivo* experiments demonstrated that SYXL ameliorated eczema-like skin lesions. Both *in vivo* and *in vitro*, SYXL inhibited the production of inflammatory cytokines, suppressed the phosphorylation of JAK2 and STAT3, thereby blocking the JAK-STAT signaling pathway.

**Conclusion:**

Our findings indicated that SYXL repaired skin barrier, suppressed inflammation, and treated PE by decreasing the generation of interleukin-1β, interleukin-6, and TNF-α, as well as inhibiting the phosphorylation of JAK2 and STAT3.

## 1 Introduction

Eczema is an inflammatory skin disorder marked by symptoms such as itching, dampness, burning, inflammatory exudation, and skin thickening (Saini and Pansare, 2019; Zhang et al., 2023). Perianal eczema (PE), a subset of eczema, is prone to acute attacks due to its unique anatomical location. If it remains unhealed for a prolonged period, it is likely to progress into a chronic condition, imposing both physical and psychological distress on patients. A survey revealed that over half (51.6%) of PE patients experienced a disease duration of 12 months or more, which significantly impacts their quality of life (Kränke et al., 2006). Currently, modern medicine has not been able to elucidate the pathogenesis of PE fully, most scholars believe that it may be related to skin barrier damage, immune disorders, diet, mental, environmental, and local stimulation (Saini and Pansare, 2019), and treatment is usually dominated by glucocorticoids and oral antihistamines (Zhang et al., 2023). However, although hormone therapy is effective in the short term, it carries several drawbacks, including drug resistance, hormone-dependent dermatitis, epidermal atrophy, and hyperpigmentation (Li et al., 2023). Additionally, antihistamines pose a risk of central nervous system suppression (Huang et al., 2023). Consequently, there exists an urgent need for PE treatments with developed safety and efficacy.

Traditional Chinese Medicine (TCM) has long recorded PE, specifically called “anal dampness”, “hydrangea style”, and “soaking sores” (An, 2005). According to TCM theory, the development of PE is primarily associated with “wind, wet, heat and insect”. TCM treatments typically include oral administration of TCM, medicinal fumigation, and external ointments (Zhang et al., 2023). Shi-Yang-Xiao lotion (SYXL hereafter), which is derived from the modification of “Kushen Decoction” in “Yang Ke Xin De Ji”, has been used for many years by Professor Zhimin Fan, a renowned TCM practitioner from Jiangsu Province, in the treatment of PE. The corresponding research is being carried out as a hospital-prepared formulation. It is composed of *Sophorae Flavescentis Radix* (Kushen in Chinese), *Phellodendri Chinensis Cortex* (Huangbo in Chinese), *Cnidii Fructus* (Shechuangzi in Chinese), *Kochiae Fructus* (Difuzi in Chinese), *Dictamni Cortex* (Baixianpi in Chinese), *Pseudolaricis Cortex* (Tujingpi in Chinese), *Saposhnikoviae Radix* (Fangfeng in Chinese) and *Alumen* (Baifan in Chinese). All herbal ingredients are listed in the Chinese Pharmacopoeia (2020 Edition).

Previous clinical studies (Tang, 2024; Chen et al., 2018) have demonstrated that SYXL is both effective and cost-efficient. When used in combination with traditional fumigation therapy, the medicinal liquid can act directly on the skin, ensuring complete absorption of active ingredients at the lesion site and thereby enhancing therapeutic efficacy.

Due to the complexity and diversity of components in TCM formulations, herbs are typically classified as monarch, minister, assistant, or guide medicines when analyzing their therapeutic roles. In SYXL, *Sophorae Flavescentis Radix* serves as the monarch herb, with functions including heat-clearing, dampness-elimination, antibacterial activity, itch relief, and anti-inflammatory (Wu et al., 2024). Its primary active compound, matrine, demonstrates diverse pharmacological activities, combining anti-inflammatory, antibacterial, antipyretic, and analgesic (Li et al., 2023). *Phellodendri Chinensis Cortex*, *Kochiae Fructus*, *Dictamni Cortex*, and *Alumen* can help *Sophorae Flavescentis Radix* enhance the efficacy of clearing heat, detoxifying, removing dampness, and relieving itch. Contemporary pharmacological investigations have demonstrated that *Dictamni Cortex* has both anti-allergic and anti-inflammatory properties (Chen et al., 2021). Therefore, they are minister medicine. *Cnidii Fructus* and *Saposhnikoviae Radix* contribute to insecticidal and antipruritic effects and help to moderate the cold nature of other herbs, serving as assistant or guide herbs. However, the active components of SYXL and its therapeutic mechanisms in PE treatment have not been fully clarified.

In this study, the primary chemical constituents of SYXL were successfully determined through the application of Ultra Performance Liquid Chromatography–Quadrupole Time-Of-Flight–Mass Spectrometry/Mass Spectrometry (UPLC-QTOF-MS/MS). In order to elucidate the potential mechanisms responsible for its therapeutic impacts on PE, we implemented network pharmacology to construct target-pathway interaction networks. Subsequently, both *in vivo* and *in vitro* experiments were carried out to verify the efficacy and mechanistic pathways of SYXL in treating PE. Furthermore, an integrated analysis of mass spectrometry data and network pharmacology results was performed to identify the underlying active ingredients of SYXL. Our research lays a theoretical groundwork for further research and development of SYXL.

## 2 Materials and methods

### 2.1 Preparation of SYXL

SYXL is composed of *Sophorae Flavescentis Radix* (Kushen in Chinese), *Phellodendri Chinensis Cortex* (Huangbo), *Cnidii Fructus* (Shechuangzi), *Kochiae Fructus* (Difuzi), *Dictamni Cortex* (Baixianpi), *Pseudolaricis Cortex* (Tujingpi), *Saposhnikoviae Radix* (Fangfeng) and *Alumen* (Baifan). All the herbs were purchased from Nanjing Hospital of Chinese Medicine affiliated to Nanjing University of Chinese Medicine. Detailed information on the herbal components of SYXL is provided in Table 1. Mix all herbs with water at a ratio of 5:1 (w/v) and boil twice, collecting the filtrate twice. The resulting filtrates were combined and lyophilized to obtain a powder for subsequent cell-based experiments.

**TABLE 1:** Ingredient list for SYXL.

Ingredients	Latin name	Plant name	Weight (g)
Kushen	*Sophorae Flavescentis Radix*	*Sophora flavescens* Ait	20
Huangbo	*Phellodendri Chinensis Cortex*	*Phellodendron chinense* Schneid	30
Shechuangzi	*Cnidii Fructus*	*Cnidium monnieri* (L.)Cuss	20
Difuzi	*Kochiae Fructus*	*Kochia scoparia* (L.)Schrad	20
Baixianpi	*Dictamni Cortex*	*Dictamnus dasycarpus* Turcz	20
Tujingpi	*Pseudolaricis Cortex*	*Pseudolarix amabilis* (Nelson)Rehd	20
Fangfeng	*Saposhnikoviae Radix*	*Saposhnikovia divaricata* (Turcz.)Schischk	15
Baifan	*Alumen*	-	30

### 2.2 UPLC-QTOF-MS/MS analysis

The sample analysis was performed *via* UPLC-QTOF-MS/MS, utilizing a SHIMADZU LC-40 liquid chromatography system coupled with a SCIEX 7600 mass spectrometer. Chromatographic separation was achieved on an InfinityLab Poroshell 120 EC-C18 column, employing a mobile phase A comprising 0.1% formic acid in water and a mobile phase B consisting of 0.1% formic acid in acetonitrile. The gradient elution profile was programmed as follows: 0–5 min, 5%–20% B; 5–20 min, 20%–30% B; 20–40 min, 30%–40% B; 40–50 min, 40%–50% B; and 50–80 min, 50%–80% B. The flow rate was set at 0.3 mL/min, with an injection volume of 5 µL. Mass spectrometric acquisition was conducted in positive and negative ion switching modes, applying spray voltages of +5500 V and −4500 V, respectively. The desolvation voltage was set to 80 V, with a collision energy of 10 V. Gas pressures were configured at 50 psi (GS1) and 35 psi (GS2) for the ion source gases, with a curtain gas pressure of 35 psi. The ion source temperature was regulated at 500°C. The mass spectrometry detection range spanned 100–1,500 Da.

### 2.3 Target prediction of SYXL by network pharmacology

The main chemical constituents and potential targets of *Sophorae Flavescentis Radix*, *Phellodendri Chinensis Cortex*, *Cnidii Fructus*, *Kochiae Fructus*, *Dictamni Cortex*, *Pseudolaricis Cortex*, *Saposhnikoviae Radix*, *Alumen* were retrieved from the Traditional Chinese Medicine molecular mechanism of bioinformatics analysis platform (BATMAN-TCM, http://bionet.ncpsb.org/batman-tcm/) and Traditional Chinese Medicine Systems Pharmacology Database (TCMSP, https://www.tcmsp-e.com/tcmsp.php). Where BATMAN-TCM analysis platform target acquisition setting conditions Prediction Score cutoff >20. We used the Uniprot database to calibrate all target names (https://www.uniprot.org/). In view of the fact that *Alumen* is mainly the product of the removal of crystal water after calcined of aqueous potassium aluminum sulfate KAl(SO_4_)_2_·12H_2_O, the 2D and 3D structures of potassium aluminum sulfate were obtained using PubChem database in this study. The obtained 2D and 3D structures were uploaded to PharmMapper (http://lilab-ecust.cn/pharmmapper/) for target prediction. PE-related disease target genes were identified from the GeneCards database using “Perianal Eczema” as the search term. The retrieved genes were exported and processed in Excel for further standardization.

### 2.4 Construction of the PPI network

The gene of SYXL active ingredient and the target gene of PE were extracted by Venny 2.1 software. The STRING database was imported with the intersecting target genes, and species parameter was set to “*Homo sapiens*” so that protein–protein interaction (PPI) network could be constructed. The “drug-component-target” network was visualized by using Cytoscape 3.7.2 software. The CytoHubba plugin was applied to analyze node degrees, and hub genes were identified based on the median degree value.

### 2.5 GO and KEGG enrichment analysis

An enrichment analysis of Gene Ontology (GO) was conducted utilizing the DAVID database, while Kyoto Encyclopedia of Genes and Genomes (KEGG) pathway enrichment analysis was executed to characterize molecular pathways. Results were visualized *via* the Bioinformatics Online Platform (http://www.bioinformatics.com.cn/), systematically categorized into biological processes (BP), cellular components (CC), molecular functions (MF), and KEGG pathways. We employed bubble plots to visually represent the top 20 key regulatory mechanisms and signaling pathways identified based on enrichment ranking.

### 2.6 Ethics and animals

All animal experiments were performed at the Laboratory Animal Center of Nanjing University of Chinese Medicine. The animal experiments have been approved by the Laboratory Animal Ethics Committee of the Nanjing University of Chinese Medicine (approval number: ACU221108).

### 2.7 Induction of eczema in rats by DNCB and drug administration

The 6-week-old Sprague-Dawley (SD) rats were randomly divided into six groups (n = 10 per group) and were fed adaptively for 1 week prior to the experiment. The perianal region of each rat was prepared before modeling. Apart from the blank group, all the other rats were treated with 2,4-Dinitrochlorobenzene (DNCB) solution (West Asia Chemical Technology Company, Shandong, China) to establish an acute PE rat model. A 7% DNCB solution was applied on day 1, followed by 5% DNCB solution on day 3, and then every 3 days thereafter, for a total of five applications. From days 18–25, the blank group and model group were given external wound washing with pure water at 37°C–40°C, while SYXL low-, medium-, and high-dose groups were treated with corresponding concentrations of SYXL solution (105, 210, 420 mg/mL per rat, twice a day, 15 min each time). The positive control group received Triamcinolone and Econazole cream (TAEC; Xi’an Janssen Pharmaceutical Company, Xi’an, China) at a dose of 21 mg/rat, applied twice daily to the perianal region. After the treatment period, all rats were anesthetized and sacrificed *via* common carotid artery blood collection. Perianal skin tissues were collected and subsequently preserved in 4% paraformaldehyde (Beijing Lanjaco Technology Company, Beijing, China) for 24 h or stored at −80°C for further analysis.

### 2.8 Measurement of scratching times

The SD rats were positioned within a transparent observation enclosure for a duration of 30 min, during which their itch - scratching behavior was monitored over the course of 1 h. Subsequently, a statistical analysis was carried out on the itch - scratching behavior exhibited by the rats in each respective group.

### 2.9 Detection of spleen and thymus index

In addition to the perianal skin, the spleen and thymus were rapidly removed during euthanasia. The spleen and thymus were measured in terms of weight in milligrams, and their respective indices were determined through the following calculation: the spleen/thymus index is obtained by dividing the mass of the spleen or thymus (expressed in milligrams) by the body mass (expressed in grams) and then multiplying the result by 10.

### 2.10 Evaluation of skin severity

The severity of skin lesions was observed daily, including erythema, papules, and scabs. The Eczema Area and Severity Index (EASI) scoring system was used for evaluation. The detailed scoring guidelines were outlined as follows: a score of zero indicated the absence of symptoms, one represented mild symptoms, two signified moderate symptoms, and three denoted severe symptoms. The sum of perianal lesions was computed by aggregating all individual scores.

### 2.11 Histologic examinations

The perianal skin of SD rats was cut into 5 μm slices after dehydration, paraffin embedding, xylene dewaxing, and washing with distilled water. Hematoxylin-eosin (HE; Nanjing Yiermei Biotechnology Company, Nanjing, China) staining was employed to evaluate the structural soundness and cellular invasion within the skin tissue of SD rats.

### 2.12 Cell culture and inflammation induction

HaCaT Cells are Human Immortalized Epidermal Cells, purchased from Sebikon Company (Shanghai, China), using 10% fetal bovine serum (Nanjing Telomere Biotechnology Company, Nanjing, China), penicillin (100U/mL), and streptomycin (100 μg/mL) (Thermo Fisher, United States) in DMEM high-glucose culture solution (Gibco, United States) cultured in a 37°C, 5% CO_2_ incubator (Thermo Fisher, United States). When the cell fusion reached 80%–90%, it was digested with trypsin (Thermo Fisher, United States) and cultured in a 1:3 ratio. HaCaT cells were harvested and inoculated on a 96-well plate at a concentration of 2 × 10^4^ cells/well, followed by a 24-h incubation period. The next day, the blank group was not given any intervention, the model group was stimulated with tumor necrosis factor-α (TNF-α, 10 ng/mL) and interferon-γ (IFN-γ, 10 ng/mL) (PeproTech, United States) for 24 h to induce cell inflammation model, and the experimental group was simultaneously treated with low-, medium- and high-concentration drug-containing medium for 24 h on the basis of modeling.

### 2.13 Detection of plasma IL-6, IL-1β, TNF-α

Blood samples obtained from SD rats were subjected to centrifugation at 3,000 rpm for 10 min at 4°C to separate the serum. Following relevant treatments, supernatants were collected. ELISA was employed to measure the concentrations of interleukin-1β (IL-1β), interleukin-6 (IL-6), and tumor necrosis factor-α (TNF-α) in both serum samples and cell culture supernatants.

### 2.14 Expression of JAK-STAT-related pathway protein by Western blot

Perianal skin tissues and HaCaT cells of rats were lysed using RIPA buffer (Beyotime, Shanghai, China) on ice for 20–30 min. The protein samples were obtained by centrifugation at 12,000 rpm for 10 min at 4°C. Subsequently, the samples underwent electrophoresis *via* sodium dodecyl sulfate-polyacrylamide gel electrophoresis (SDS–PAGE; Yeasen, Shanghai, China) and were transferred onto a polyvinylidene fluoride (PVDF) membrane (Millipore, United States). The membranes were blocked with 5% skim milk in Tris-buffered saline containing 0.1% Tween-20 (TBST) for 30 min at room temperature. Primary antibodies targeting STAT3, p-STAT3, JAK2, p-JAK2, and β-actin (CST, Boston, United States) were incubated overnight at 4°C, followed by IgG anti-mouse or anti-rabbit antibodies conjugated to horseradish peroxidase (Abcam, UK) at room temperature for 1 h. Protein bands were visualized with an enhanced chemiluminescence (ECL) detection reagent (Beyotime, Shanghai, China) and captured using a gel documentation system (Clinx, Shanghai, China). Quantitative analysis was conducted utilizing Clinx Image Analysis software (Clinx, Shanghai, China).

### 2.15 Statistical analysis

Data are expressed as the mean ± standard deviation. All statistical analyses were performed employing GraphPad Prism v8.01 (GraphPad Software, La Jolla, CA, United States). For multiple comparisons, one-way analysis of variance (ANOVA) was conducted, and subsequent *post hoc* analysis was performed using Dunnett’s test. A p-value of <0.05 was considered statistically significant.

## 3 Results

### 3.1 Identification of active components in SYXL

**FIGURE 1:**
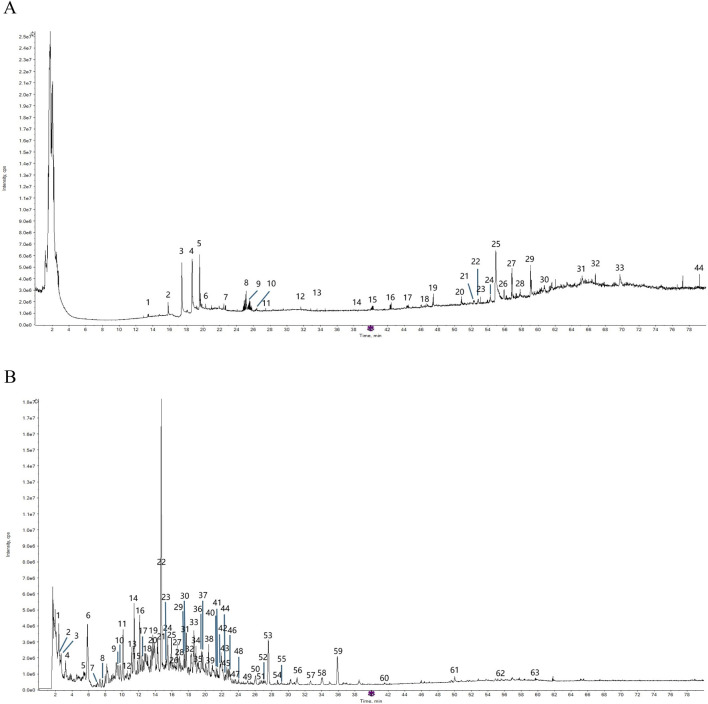
The TIC of SYXL was collected by UPLC-QTOF-MS/MS. **(A)** Positive mode. **(B)** Negative mode.

The total ion chromatogram (TIC) of SYXL was obtained using UPLC-QTOF-MS/MS technique (Figure 1), from which 93 compounds were identified, including kurarinol, sophoraflavoside II, dictamine, *etc.* Subsequently, mass spectrometry results were combined with network pharmacology analysis, yielding 10 common active ingredients such as matrine, sophoridine, quercetin, and berberine. The chemical structures and molecular formulas of these compounds are summarized in Table 2.

**TABLE 2:** Ten active chemical components in SYXL were identified through a combined analysis of network pharmacology and UPLC-QTOF-MS/MS. Chemical structure obtained from PubChem.

Num	Rt/min	Identified component	Formula	Cas#	Chemical structure	Drug
1	19.02	Matrine	C15H24N2O	519–02-8	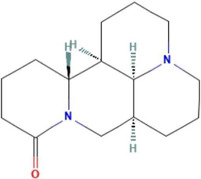	Sophorae Flavescentis Radix
2	19.25	Sophoridine	C15H24N2O	6,882–68-4	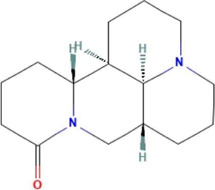	Sophorae Flavescentis Radix
3	22.46	Quercetin	C15H10O7	117–39-5	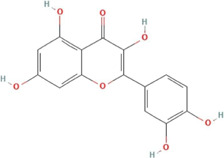	Dictamni Cortex; Phellodendri Chinensis Cortex; Sophorae Flavescentis Radix
4	6.14	Berberine	C20H18NO4	2086–83-1	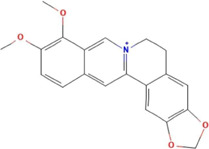	Phellodendri Chinensis Cortex
5	15.17	Thalifendine	C19H16NO4	18,207–71-1	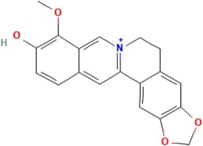	Phellodendri Chinensis Cortex
6	15.31	Coptisine	C19H14NO4	3,486–66-6	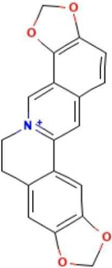	Phellodendri Chinensis Cortex
7	21.2	Berberrubine	C19H16ClNO4	15,401–69-1	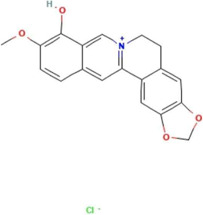	Phellodendri Chinensis Cortex
8	16.21	Kushenol E	C25H28O6	99,119–72-9	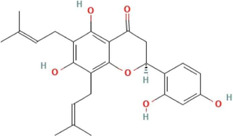	Sophorae Flavescentis Radix
9	13.05	Kurarinone	C26H30O6	34,981–26-5	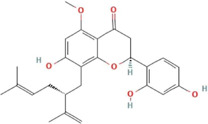	Sophorae Flavescentis Radix
10	54.52	Geranylacetone	C13H22O	68,228–05-7	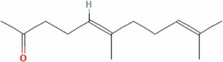	Saposhnikoviae Radix

### 3.2 Network pharmacology

#### 3.2.1 Overlapped targets analysis

**FIGURE 2:**
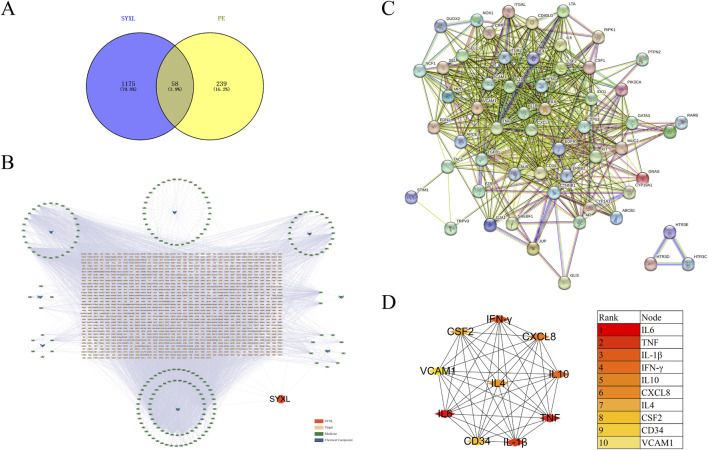
Analysis of relevant target genes of SYXL and PE. **(A)** Venny diagram. **(B)** Target interaction network. **(C)** PPI network. **(D)** Top 10 target plots.

Network pharmacology was employed to forecast the potential therapeutic targets of SYXL, thereby laying a theoretical foundation for elucidating its molecular mechanism in the management of PE. Through systematic database screening (TCMSP, BATMAN-TCM, UniProt, GeneCards), we compiled 1,175 potential therapeutic targets related to drug components and 239 targets associated with PE pathogenesis (Figure 2A). Among these, 58 overlapping genes were identified as potential therapeutic targets of SYXL for PE treatment.

#### 3.2.2 PPI network analysis

**FIGURE 3:**
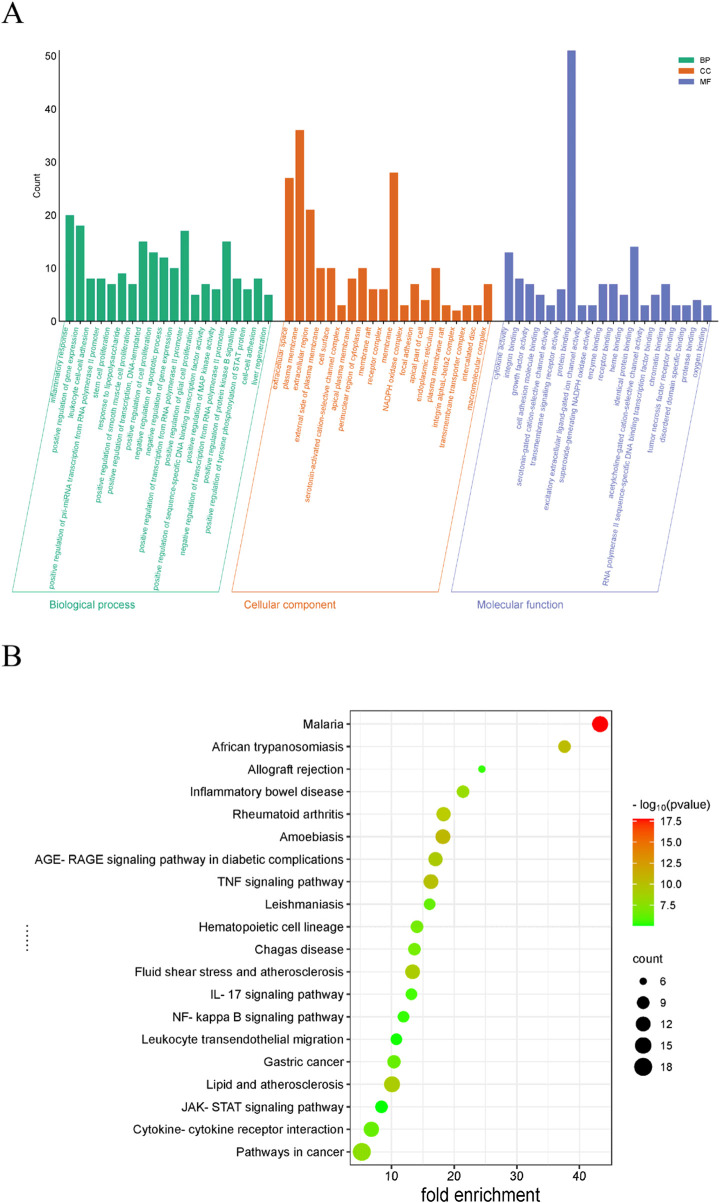
GO functional enrichment and KEGG pathway analysis. **(A)** GO analysis. **(B)** KEGG analysis.

After removing one isolated gene, a PPI network map of component targets, including 57 overlapping target genes, was constructed using the cross genes of SYXL and PE (Figures 2B, C). The top 10 hub targets identified were IL-6, TNF, IL-1β, IFN-γ, IL-10, CXCL8, IL-4, CSF2, CD34 and VCAM1 (Figure 3D). Among them, IL-6, IL-1β, and TNF showed the highest degrees, suggesting their probable significance in SYXL’s anti-PE mechanisms.

#### 3.2.3 Functional and pathway enrichment assessment of SYXL

GO analysis identified 262 BP items, 33 CC items, and 40 MF terms with *p* < 0.05. In Figure 3A, the top 20 terms with the most pronounced statistical enrichment in each category are presented, such as inflammatory response. KEGG pathway analysis yielded 178 significantly enriched pathways (*p* < 0.05). In the bubble plot (Figure 3B), the top 20 pathways included several inflammation-related signaling pathways, among the Janus kinase (JAK)/signal transducer and activator of transcription (STAT).

### 3.3 Animal experiments and efficacy verification

#### 3.3.1 Effect of SYXL on DNCB-induced PE rats skin

**FIGURE 4:**
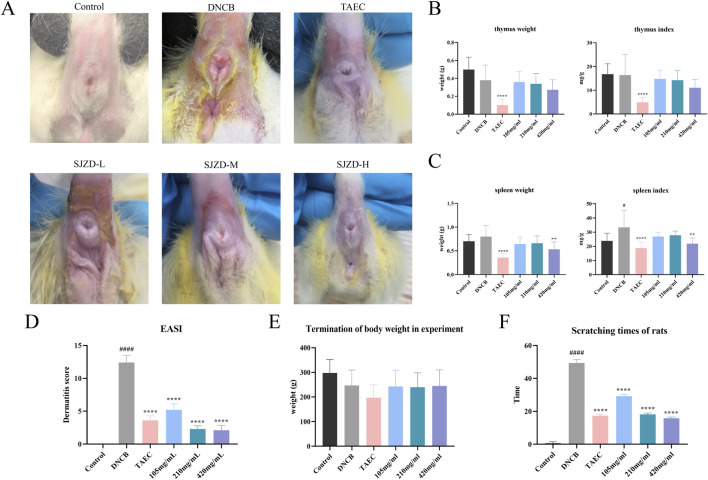
**(A)** Comparison of skin lesion improvement after SYXL treatment. **(B)** Spleen weight and spleen index results. **(C)** Thymus weight and thymus index results. **(D)** EASI skin lesion score. **(E)** The body weight after treatment. **(F)** Scratching times of rats during treatment. All data were expressed as mean ± standard deviation (n = 10), #*p* < 0.05 compared with the blank group; Compared with the model group, ****p < 0.0001, **p < 0.01.

**FIGURE 5:**
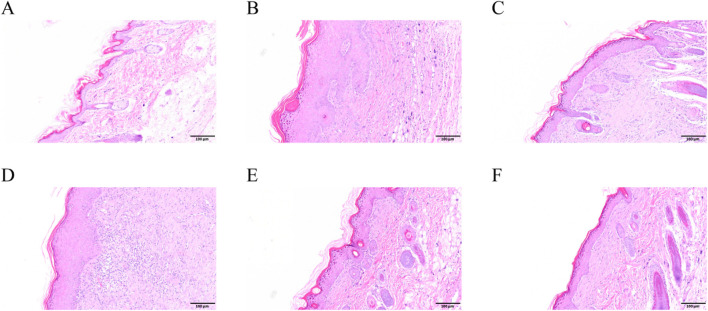
HE results of SD rats. **(A–F)** Control, DNCB, TAEC, SYXL group 105 mg/mL, SYXL group 210 mg/mL, SYXL group 420 mg/mL.

Perianal skin lesions were assessed at the experimental endpoint to evaluate the therapeutic efficacy of SYXL against PE systematically (Figure 4A). Differing from the blank group, the model group displayed pronounced skin damage, including erythema, edema, exudation, and scab formation, accompanied by a significant increase in EASI scores (*p* < 0.0001). After treatment with SYXL (105, 210, 420 mg/mL), skin scabs, papules, redness, and swelling were significantly reduced, and the extent of skin lesion recovery demonstrated a dose-dependent relationship with the escalating dosage of SYXL, and the efficacy of SYXL (210, 420 mg/mL) was superior to that of TAEC group (Figure 4D). The thymus index results revealed that the model group exhibited a lower value compared to the blank group. In contrast, the thymus index of the SYXL treatment group with different doses was close to the blank group, and the result was better than that of the positive drug group (Figure 4B). The spleen index of SD rats was notably elevated in the model group relative to the blank group, while different doses of SYXL treatment markedly reduced the spleen index, which was better than that in the positive group (Figure 4C). As illustrated in Figure 4E, the body weight of SD rats in model group was found to be significantly reduced compared to that of rats in the blank group. After SYXL intervention, the body weight exhibited an upward tendency, yet the difference did not reach statistical significance (*p* > 0.05). The therapeutic efficacy of experimental drugs was evaluated by comparing scratching times pre- and post-treatment. Figure 4F shows that scratching frequency after administration is significantly reduced in comparison to the model group, with 420 mg/mL group exhibiting a more pronounced effect than the positive drug group. In order to further assess the effect of SYXL on perianal skin of SD rats, histopathological analysis was performed. HE staining revealed that the model group displayed epidermal structural disruption, extensive hyperkeratosis, dermal papillary edema, and inflammatory cell infiltration. SYXL treatment attenuated these pathological changes, particularly in the 420 mg/mL group, which exhibited only mild epidermal thickening and notably reduced dermal inflammatory infiltration (Figures 5A–F). These findings indicate that SYXL can effectively alleviate DNCB-induced skin inflammation.

#### 3.3.2 Influence of SYXL on the concentrations of inflammatory cytokines in SD rat serum

**FIGURE 6:**
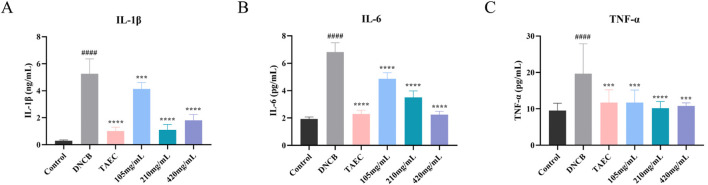
Influence of SYXL on the secretion of inflammatory factors. **(A-C)** IL-1β, IL-6, and TNF-α. All data were expressed as mean ± standard deviation (n = 10). Compared with the blank group, ^####^ *p* < 0.0001; Compared with the model group, ^****^ *p* < 0.0001, ^***^ *p* < 0.001.

Network pharmacology analysis suggested that the therapeutic mechanism underlying SYXL’s treatment of PE might entail the suppression of inflammatory signaling pathways, wherein IL-6, IL-1β, and TNF were identified as pivotal targets. To validate these findings, ELISA was employed to measure the serum concentrations of IL-6, IL-1β, and TNF-α in rats (Figures 6A–C). SYXL treatment at concentrations of 105, 210, and 420 mg/mL effectively reduced the expression of these cytokines, confirming its anti-inflammatory effect.

#### 3.3.3 Effect of the SYXL on protein expression in SD rats

**FIGURE 7:**
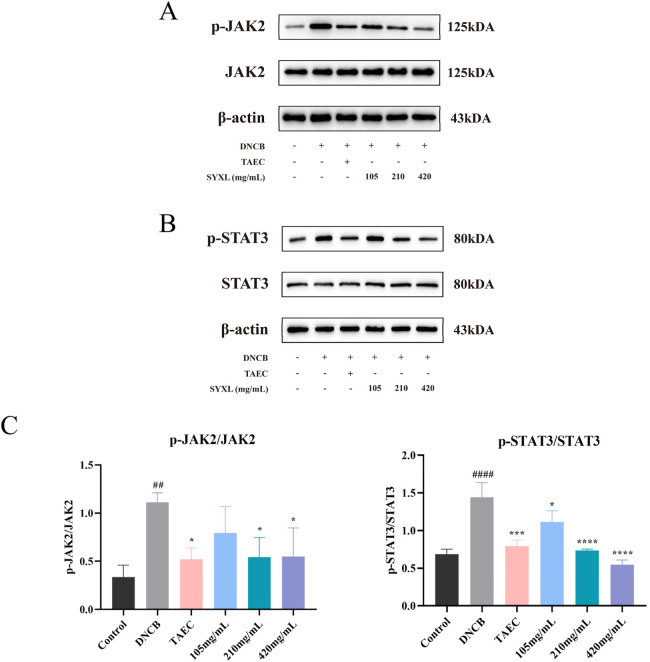
SYXL inhibited the JAK-STAT pathway in SD rats. **(A)** Western blotting detected the expression levels of JAK2 and p-JAK2. **(B)** Expression of STAT3 and p-STAT3. **(C)** Expression of p-JAK2/JAK2 and p-STAT3/STAT3. All the data were expressed as mean ± standard deviation (n = 3). Compared with the blank group, ####p < 0.0001, ##p < 0.01; Compared with the model group, ****p < 0.0001, ***p < 0.001, *p < 0.05.

To verify the network pharmacological prediction, we conducted a Western blotting experiment. As illustrated in Figure 7, the phosphorylation levels of JAK2 and STAT3 were notably elevated in DNCB-induced rat skin tissue relative to the blank group. SYXL treatment markedly reduced the phosphorylation of JAK2 and STAT3, suggesting that the effect of SYXL on PE rats might be associated with the inhibition of JAK2 and STAT3 phosphorylation levels.

### 3.4 Cell experiments and efficacy verification

#### 3.4.1 SYXL inhibits the generation of inflammatory factors in HaCaT cells induced by TNF-α/IFN-γ

**FIGURE 8:**
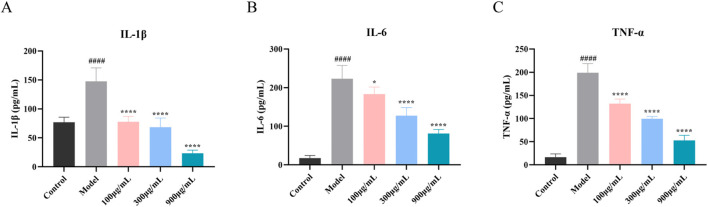
Influence of SYXL on the secretion of inflammatory factors. **(A-C)** IL-1β, IL-6, and TNF-α. All data were expressed as mean ± standard deviation (n = 6). Compared with the blank group, ####*p* < 0.0001; Compared with the model group, ****p < 0.0001, *p < 0.05.

For the purpose of evaluating the protective efficacy of SYXL on HaCaT cells subjected to TNF-α/IFN-γ-induced inflammatory damage, we collected the cell supernatant after SYXL intervention (100, 300, 900 μg/mL). ELISA kits were employed to quantify the production of three crucial inflammatory cytokines (IL-6, IL-1β, and TNF-α), which had been forecasted by network pharmacology (Figures 8A–C). The findings exhibited that SYXL significantly downregulated the expression of three cytokines in a manner that was dependent on the dosage.

#### 3.4.2 SYXL suppressed JAK-STAT pathway in TNF-α/IFN-γ-induced HaCaT cells

**FIGURE 9:**
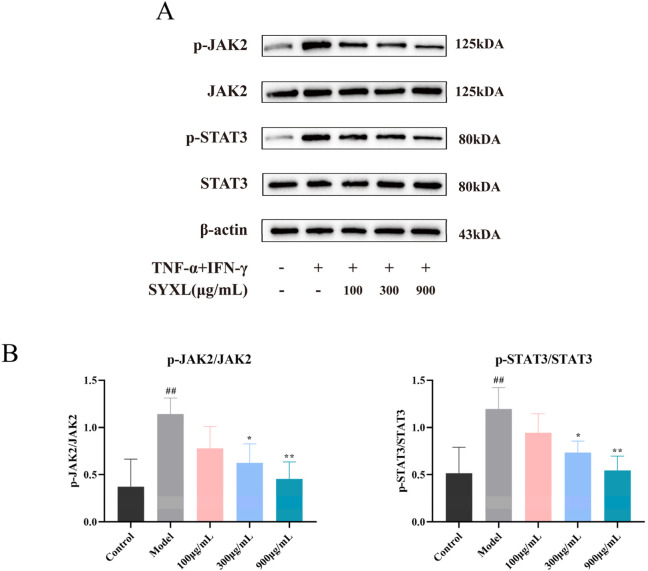
SYXL inhibited the JAK-STAT pathway in HaCaT cell. **(A)** Western blotting detected the expression levels of JAK2, p-JAK2, STAT3, and p-STAT3. **(B)** Expression of p-JAK2/JAK2 and p-STAT3/STAT3. All the data were expressed as mean ± SD (n = 4), and compared with the blank group, ^##^ *p* < 0.01; Compared with the model group, **p < 0.01, *p < 0.05.

To further verify the impact of SYXL on the JAK-STAT pathway in HaCaT cells, the protein levels of JAK2, p-JAK2, STAT3, and p-STAT3 were examined. As depicted in Figure 9, TNF-α/IFN-γ co-stimulation markedly increased the phosphorylation of JAK2 and STAT3 in HaCaT cells when compared with blank group. In contrast to the model group, SYXL (300, 900 μg/mL) inhibited the phosphorylation of JAK2 and STAT3, suggesting that suppression of JAK/STAT pathway activation may represent a key mechanism underlying SYXL’s anti-inflammatory and anti-eczema properties.

## 4 Discussion

Eczema represents a complex inflammatory dermatosis, clinically manifesting as polymorphic lesions with frequent exudative components (Huang et al., 2023), which can occur in any part of the human body. Due to the unique anatomical and physiological features of the anal region, PE tends to be more severe and persistent (Weyandt et al., 2020). The skin around the anus is in a humid environment for a long time, and this moist state is conducive to the reproduction and growth of microorganisms, coupled with the continuous discharge of physiological secretions, which has a stimulating effect on the skin and is easy to aggravate local skin damage. Although topical corticosteroids have been shown to provide short-term symptomatic relief, their long-term use is associated with notable local and systemic side effects, such as skin atrophy and capillary dilation (Li et al., 2017).

**FIGURE 10:**
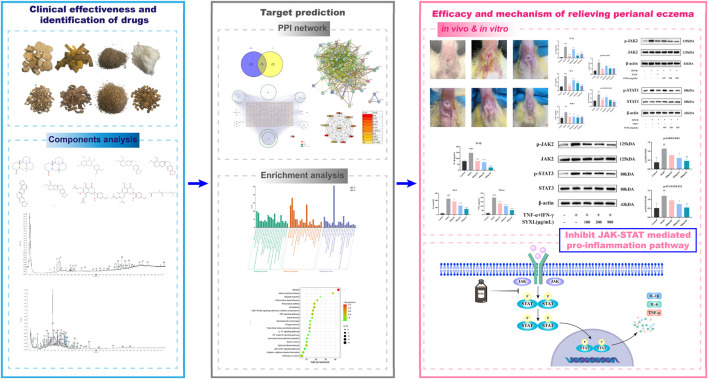
The molecular mechanism of treating PE by SYXL was summarized. By inhibiting the expression of IL-6, IL-1β, and TNF-α, inhibiting the phosphorylation of JAK2 and STAT3, and interfering with the JAK-STAT-mediated pro-inflammatory signaling pathway, SYXL achieved the purpose of treating PE.

TCM is considered a promising alternative for the treatment of PE due to its favorable efficacy and safety profile (Wang et al., 2022; Wang et al., 2021). Accumulating clinical evidence demonstrates that numerous herbal medicines and TCM formulations exhibit significant efficacy in eczema management, particularly in reducing inflammation and pruritus, such as Huanglian ointment and Mongolian herbal medicine Cymbaria daurica L (Huang et al., 2023; Wang et al., 2022). There remains a lack of TCM preparations specifically developed for PE. SYXL, a TCM compound used clinically for over 30 years, has shown significant therapeutic efficacy in previous studies. Compared with corticosteroids, SYXL offers advantages in drug safety and recurrence prevention (Tang, 2024). In addition, the administration method is to directly use liquid medicine to clean the affected area, avoiding the skin-sealing injury easily caused by the thick texture of cream drugs. Simultaneously, it is capable of playing a specific role in cleaning the diseased skin. Despite its long clinical use, the underlying mechanism of SYXL in treating PE remains unclear. This study aimed to evaluate its therapeutic effects in a DNCB-induced PE rat model and TNF-α/IFN-γ co-stimulated HaCaT cell model, while exploring its potential mechanisms of action (Figure 10).

In this research, UPLC-QTOF-MS/MS was employed to identify 93 chemical constituents in SYXL, providing a material basis for subsequent investigation of its therapeutic mechanisms in PE. Network pharmacology analysis predicted 58 overlapping target genes associated with both SYXL and PE. By integrating UPLC-QTOF-MS/MS data with network pharmacology results, 10 common active compounds were identified, including matrine, sophoridine, quercetin, berberine, thalifendine, coptisine, berberrubine, kushenol E, kurarinone and geranylacetone, and their chemical structures were determined.

Previous studies have demonstrated that matrine and berberine can suppress inflammatory factors such as TNF-α and downregulate key genes in inflammatory pathways, thereby exerting therapeutic effects on eczema (Lin et al., 2023). As the primary bioactive alkaloid in *Sophorae Flavescentis Radix*, matrine ameliorates skin pathology in eczema mouse models by reducing STAT3 mRNA levels, contributing to its anti-eczema activity (Maskey et al., 2024). Similarly, sophoridine and kurarinone inhibit inflammatory cytokine production and modulate signaling pathways, exhibiting anti-inflammatory and immunomodulatory properties (Wang et al., 2025; Gu et al., 2025). In atopic dermatitis models, quercetin shows potential as a targeted therapy (Hou et al., 2019), while coptisine alleviates inflammation by suppressing the TXNIP/NLRP3 inflammasome (Li et al., 2024). The therapeutic potential of thalifendine, berberrubine, kushenol E, and geranylacetone in eczema and other skin diseases requires further validation.

Although our study evaluated the overall efficacy of SYXL in treating eczema, the specific contributions of its active components remain unverified. Existing evidence supports the therapeutic effects of *Sophorae Flavescentis Radix* and its alkaloids, such as matrine and oxymatrine, on skin diseases (Zhang et al., 2020). For instance, drug combinations containing *Sophora flavescens*–*Angelica sinensis* inhibit the TLR4/MyD88/NF-κB pathway to treat eczema (Sun et al., 2024). Notably, SYXL contains high doses of *Sophorae Flavescentis Radix* and *Phellodendri Chinensis Cortex*, and their active alkaloids (matrine and berberine) exhibit synergistic antibacterial effects *in vitro* (Meng et al., 2024). Future studies will focus on elucidating the mechanistic roles of *Sophorae Flavescentis Radix*–*Phellodendri Chinensis Cortex* combinations (particularly matrine and berberine) in SYXL-mediated eczema therapy.

Ulteriorly, the PPI network analysis proved that the core targets were closely associated with inflammatory response. Excessive activation of these inflammatory mediators is a hallmark of skin barrier dysfunction-related diseases (Nedoszytko et al., 2014). Results from KEGG analysis indicated that the most significantly enriched pathways were predominantly associated with infectious diseases and hematologic disorders. Notably, several pathways implicated in inflammatory skin disorders were enriched, including JAK-STAT, AGE-RAGE, TNF, IL-17, and NF-κB pathways.

The JAK-STAT pathway serves as a quintessential signaling transduction mechanism for diverse cytokines factors. JAK2, a key intracellular kinase, mediates signaling for cytokines including IL-6 and IL-10 (Zeng et al., 2022). STAT3 functions as a central orchestrator of cellular immunity and inflammatory responses, orchestrating diverse biological events including cell proliferation, differentiation, and apoptosis (Silva-Carmona et al., 2020). This pathway is essential for maintaining both innate and adaptive immunity (Ahmad et al., 2017) and is critically involved in inflammatory processes (Zeng et al., 2022), becoming an emerging direction in dermatology. Prior investigations have confirmed that inhibition of JAK and STAT phosphorylation can effectively alleviate the progression of atopic dermatitis (Li et al., 2023).

Currently, *in vivo* models of PE are relatively rare. DNCB is a widely used hapten for inducing contact dermatitis. The most common use of DNCB is for the establishment of animal models of eczema (Kim et al., 2021). Based on this, we established a PE rat model using DNCB and confirmed the successful construction of the model through morphological observation, HE staining, spleen index evaluation, serum cytokine analysis, and other experiments. Subsequent pharmacological intervention experiments demonstrated that SYXL exerted significant therapeutic effects in PE rats. ELISA and Western blotting analyses revealed that SYXL alleviated eczema-like skin lesions induced by DNCB in animal models, reversed histopathological abnormalities, curbed the expression of pro-inflammatory cytokines IL-1β, IL-6, and TNF-α, and inhibited the phosphorylation of JAK2 and STAT3. In this study, SYXL was administered through a TCM fumigation method, in which the decoction was heated and applied by fumigating and soaking the perianal area. This administration method not only cleaned the wound and softened keratin, but also promoted transdermal absorption, reduced local irritation, and accelerated the removal of necrotic tissue, thereby enhancing wound healing.

Finally, an inflammatory keratinocyte model was established by co-stimulating HaCaT cells with TNF-α and IFN-γ. The generation of IL-1β, IL-6, and TNF-α was effectively suppressed by SYXL treatment. Western blotting results provided further validation, demonstrating that the anti-PE effects of SYXL are primarily achieved by inhibiting the JAK-STAT mediated inflammatory pathway. Overall, our study demonstrated consistent findings across network pharmacology, as well as *in vivo* and *in vitro* experimental investigations. SYXL exerts its therapeutic impacts on PE through the attenuation of JAK2 and STAT3 phosphorylation, thereby blocking JAK-STAT pathway activation, reducing inflammation, and promoting skin barrier repair.

This study has several limitations. First, we did not conduct a comprehensive analysis of all the active ingredients identified. Future research should further characterize these constituents and investigate their roles in the treatment of PE. Additionally, this study primarily focused on the JAK-STAT signaling pathway. Future studies should explore additional signaling pathways that may contribute to SYXL’s therapeutic efficacy to understand its pharmacological mechanisms comprehensively.

## 5 Conclusion

This research confirmed the therapeutic efficacy of SYXL in curing PE and explored its underlying mechanisms, providing new insights into the pharmacological evaluation of TCM compounds. Our findings highlight that SYXL exerts its effects primarily by reducing inflammatory cytokine expression and inhibiting JAK2 and STAT3 phosphorylation. The robust efficacy demonstrated in this study, such as alleviating itching and repairing skin damage, positions SYXL as a hopeful candidate for subsequent drug development and clinical implementation. Subsequent steps could involve further screening of its effective drug components, optimizing the formulation, and conducting multi-center clinical trials to further confirm the effective drug composition and clinical efficacy of SYXL.

## Data Availability

The original contributions presented in the study are included in the article/supplementary material, further inquiries can be directed to the corresponding author.

## Abbreviations

BP, biological processes; CC, cellular components; DNCB, 2,4-Dinitrochlorobenzene; EASI, Eczema Area and Severity Index; ECL, enhanced chemiluminescence; GO, Gene Ontology; HE, Hematoxylin‐eosin; IFN-γ, Interferon-γ; IL‐17, interleukin‐17; IL-1β, interleukin-1β; IL‐6, interleukin-6; JAK, Janus kinase; KEGG, Kyoto Encyclopedia of Genes and Genomes; MF, molecular functions; NF-κB, nuclear factor-κB; PE, Perianal Eczema; PPI, protein–protein interaction; PVDF, polyvinylidene fluoride; SD, Sprague-Dawley; SDS–PAGE, sodium dodecyl sulfate-polyacrylamide gel electrophoresis; STAT, signal transducer and activator of transcription; SYXL, Shi‐Yang-Xiao lotion; TAEC, Triamcinolone and Econazole cream; TBST, Tris‐buffered saline with 0.1% Tween-20; TCM, Traditional Chinese Medicine; TIC, total ion chromatogram; TNF‐α, tumor necrosis factor‐α; UPLC-QTOF‐MS/MS, Ultra Performance Liquid Chromatography‐Quadrupole Time-Of‐Flight‐Mass Spectrometry/Mass Spectrometry.
